# Descriptive study of triple negative breast cancer in Eastern Algeria

**DOI:** 10.11604/pamj.2018.29.45.12523

**Published:** 2018-01-18

**Authors:** Haddad Souad, Frimeche Zahia, Lakehal Abdelhak, Sifi Karima, Satta Dalila, Abadi Noureddine

**Affiliations:** 1 Higher National School of Biotechnology, Constantine, Algeria; 2 Laboratory of Biology and Molecular Genetic, Constantine 3 University, Algeria; 3 General Surgery Service, Regional Hospital, Constantine, Algeria; 4 Epidemiology Service, CHU Ibn Badis, Constantine, Algeria; 5 Cellular and Molecular Biology Laboratory, Constantine 1 University, Algeria

**Keywords:** Triple negative breast cancer, epidemiology, clinocopathologic features, overall survival, Eastern Algeria

## Abstract

**Introduction:**

Triple-negative breast cancer (TNBC) is characterized by the lack of estrogen receptor, progesterone receptor and human epidermal growth factor receptor-2 (HER-2) expression. It is aggressive and most common in African women. In this study we identified the frequency, clinical an pathological characteristics of this type in a cohort of women in Eastern Algeria.

**Methods:**

We conducted a retrospective study between January 2010 and December 2015 at the regional hospital of Constantine in eastern Algeria. Among 472 women with breast cancer, 102 women had a TNBC. Clinical and pathological features and overall survival were analyzed.

**Results:**

21.61% of patients had TNBC with a median age of 52 years. 65.31% of the patients were menopausal. Only 6.82% of women had a family history of breast cancer. The majority of patients had infiltrating ductal carcinoma (96.08%), the proportion of grade III SBR was 53.92%. The average tumor size was 4.4cm and 70.1% of the tumors had a very large size (T2 and T3). 69.07% of patients had positive lymph nodes, vascular invasion was found in 48.57% of cases. Metastatic sites were bone in 16.13% of cases, hepatic in 3.26% and pulmonary in 0.98%. For treatment modalities, 95.24% underwent surgery and adjuvant chemotherapy. 33.33% of patients have received neoadjuvant chemotherapy with 29% of complete pathologic response (pCR) and 96.97% have received radiotherapy. Overall Survival (OS) for all patients at 5 years was 45.2%.

**Conclusion:**

Most of our results are in accordance with literature data, however we noted some discrepancies. In this study, TNBC is more common in menopausal women than non menopausal women and characterized by a low rate of visceral metastases and a lower overall survival at 5 years.

## Introduction

Breast cancer is a major public health problem. In 2014, 10910 new cases of breast cancer were diagnosed in Algeria [1]. Since1990, it became more frequent than cervix cancer. Indeed, the incidence increased from 14.5 new cases per 10 ^5^ inhabitants in 1993 to 70.2 per 10 ^5^ in 2012 [2]. Triple-negative breast cancer (TNBC) is a heterogeneous group characterized by the absence of expression of hormone receptors (HR) to estrogen, progesterone and human epidermal growth factor receptor-2 (HER-2) [3]. It accounts for 12 to 17% of all breast cancers [4]. This percentage depends on the methods used to define the threshold of positivity and negativity of HR and HER2 expression. The definitions of the TNBC by immunohistochemistry are discordant, for certain definitions the threshold of positivity of the RH is more than 10%. For other definitions, the threshold of negativity retained is less than 1% of tumor cells expressing the HR [5]. TNBC occurs most frequently in young women under the age of 50 and are more common among women of African origin [6, 7]. TNBC is associated with a high risk of metastasis and death, mainly during the first 5 years of follow-up. Metastatic progression is characterized by early relapse, with predominance of visceral and cerebral localizations and a lower incidence of bone metastases [8]. The purpose of this descriptive study, carried out for the first time in Algeria, is to determine the frequency of TNBC and to identify its clinical and pathological characteristics in the eastern Algerian region.

## Methods

**Study population**: We undertook a retrospective study between January 2010 and December 2015. This study involved 472 files of women with breast cancer, treated and monitored in the oncology service of the regional hospital in Constantine. 102 women were identified as having TNBC, aged 25 to 85 years, from 15 provinces of eastern Algeria. The thresholds of negativity retained are less than 10% of cells labeled for hormonal receptors and a HER2 membrane labeling of score 0 or +1 or 2+ without gene amplification in FISH (fluorescence in situ hybridization technique).

**Data collection**: The data collected included age, histological tumor type, tumor grade according to Scarff Bloom and Richardson histological system, tumor size, vascular invasion, lymph node invasion, TNM classification of tumors, bone metastases, visceral metastases and treatment. Data is entered on Microsoft Office Excel 2013 and analyzed using SPSS software Version 20. Overall Survival (OS) rate was estimated by Kaplan and Meier analysis.

## Results

**Figure 1:**
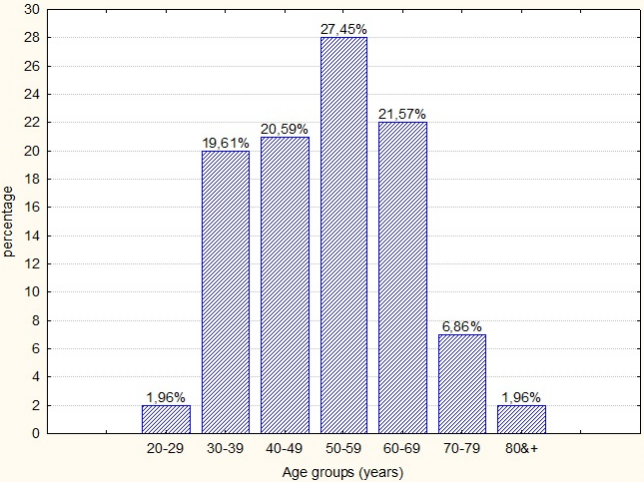
Distribution of patients according to age

**Characteristics of the population study**: Among 472 cases of breast cancer, 102 women (21.61%) had TNBC. The age of the patients varied from 25 to 85 years. The average age of patients was 51.94 ± 13.02 years; the median age was 52 years. The distribution of the population according to age showed that the predominant age group was that between 50-59 years (27.47%) (Figure 1). 65.31% of the patients were menopausal. Only 6.82% of women had a family history of breast cancer. The histological study showed that the predominant histological type was infiltrating ductal carcinoma in 96.08% of cases, infiltrating lobular carcinoma represented 2.94%, a single infiltrating papillary carcinoma was recorded at 0.98%. According to grading Scarff Bloom and Richardson histological system, the proportion of grade III (poor prognosis) was estimated at 53.92% and that of grade II (intermediate prognosis) was 45.10% while the proportion of grade I (good prognosis) was 0.98 %. The average tumor size was 4.4cm with extremes ranging from 1cm to 11cm. According to the TNM classification, 6.19% of the tumors were classified T1, 47.42% were classified T2, 22.68% were classified T3 and 23.71% were classified T4. For the lymph node involvement, 69.07% had positive lymph nodes, 29.90% were classified (N1), 26.80% were (N2) and 12.37% were (N3). Vascular invasion was found in 48.57% of cases. At diagnosis, 16.33% of the patients were metastatic. Metastatic sites were bone in 16.13% of cases, hepatic in 3.26% and pulmonary in 0.98% (only one case) (Table 1).

**Table 1:** Clinical and pathological characteristics of patients

Variable	Frequency
TNBN	21.61%
Median age	52 years
**Menopause**	
Yes	65.31%
No	34.69%
**Family history**	
Yes	6.82%
No	93.18%
**Histological type**	
Infiltrating ductal carcinoma	96.08%
Infiltrating lobular carcinoma	2.94%
Infiltrating papillary carcinoma	0.98%
**SBR gradding**	
I	0.98%
II	45.10%
III	53.92%
Average tumor size	4.4cm
T1	6.19%
T2	47.42%
T3	22.68%
T4	23.71%
Vascular invasion	48.57%
Positive lymph nodes	69.07%
Negative lymph nodes	30.93%
Distant metastasis	16.33%
**Metastatic sites**	
Bone	16.33%
Liver	3.26%
Lung	0.98%

**Treatment modalities**: For treatment modalities, all patients underwent Patey type mastectomy. Patients with operable tumors (95.24%) underwent surgery and adjuvant chemotherapy. Patients with no operable tumors (33.33%) have received neoadjuvant chemotherapy followed by surgery; the rate of complete pathologic response (pCR) was 29%. 96.97% of patients have received radiotherapy (Table 2).

**Table 2:** Treatment modalities

Patey type mastectomy	100%
Chemotherapy	
Adjuvant	95.24%
Neoadjuvant	33.33%
pCR	29%
Radiotherapy	96.97%

**Figure 2:**
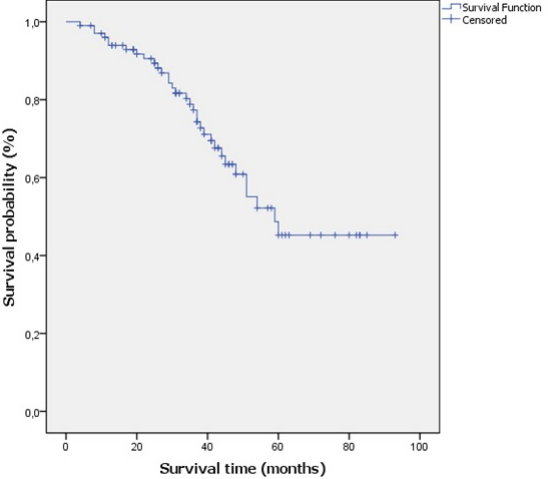
Overall survival of all patients at 5 years

**Overall survival**: After having collected the telephone numbers of the patients, we contacted them or their cousins, 33 women had died (32.4%), 47 women were alive (46.1%) and 22 were lost to sight (21.6%). The Overall Survival (OS) of all patients is the time between the date of diagnosis (date of biopsy) and death or last follow-up. We did note evaluate the effect of treatment on survival. OS at 5 years for all patients was 45.2% (Figure 2).

## Discussion

The frequency of TNBC is poorly known in Algeria. The frequency reported in this study (21.61%) is higher than those reported in Morocco [9-12]. These studies report frequencies of 17.5%, 16.5%, 11.3% and 17.6% respectively. In the United States, the frequency of the CSTN is 20% [13]. In Canada, it is 11.2% [14]. In Pakistan, the CSTN is diagnosed in 36.7% of patients with breast cancer [15]. In Mexico, it represents 23.1% [16]. The frequency of the TNBC varies according to the threshold of negativity fixed for HR and HER2 and ethnicity. This frequency is higher among women of African and Hispanic descent [6, 17]. Studies on the TNBC [12, 14, 16, 18] show that this type most often affects young and non-menopausal women, the median age of diagnosis varies from one study to another (40 years to 53 years). In our study the median age at diagnosis was 52 years, it is higher than that of Moroccan women [9, 10, 12] and close to that of American women [14] and Hispanic women [16]. In this study, menopausal women represent 65.31% of patients, which is not concordant with the studies cited previously. This could be due to tardy diagnosis of cancer since most patients have large tumors. In this study, six women (6.82%) had a family history of breast cancer. This result is close to that reported by Rais et al [10]. The relationship between the mutations of the BRCA1 gene and the development of TNBC is well defined. Women with breast cancer associated with a germline mutation of BRCA-1 usually present with a tumor of triple-negative phenotype [19, 20]. In the absence of family history, sporadic mutations of the BRCA1 gene are detected in women with TNBC [21]. The proportion of triple negative tumors exhibiting a sporadic mutation of BRCA1 is about 40%. The majority of sporadic CSTN cases are characterized by inactivation of the BRCA1 gene due to epigenetic modifications such as methylation [22]. A study of the BRCA1 gene for our patients will be undertaken. Triple negative carcinomas are mainly high-grade ductal carcinomas [9, 10, 14, 23, 24]. The results we obtained are in agreement with the literature, infiltrating ductal carcinoma is the most frequent (96.08%) and most tumors are of high grade (53.92%). However, there are rare triple-negative tumors with better prognosis: cystic adenoid tumors, apocrine tumors, secretory tumors and metaplastic tumors [25]. In this study, none of these histological types was reported.

Triple-negative tumors are usually large (> 2 centimeters in diameter at diagnosis in 2/3 of cases) [14], this is due to a rapid growth of these tumors so that these tumors escape from screening by mammography [26], their size varies from 3 to 3.6 cm [12, 14]. In this study, the average tumor size was very large (4.4cm) and 70.1% of the tumors had a very large size (T2 and T3). We suggest that patients were diagnosed tardily. The risk of lymph node involvement is poorly defined, for some studies [12, 27] there is no correlation between tumor size and lymph node infiltration. For other studies [9, 14], the risk of node invasion increases with increasing tumor size, which is consistent with our results where 69.07% of the lymph nodes were infiltrated. In the TNBC, compared to other breast cancer groups, the risk of distant metastasis is lower in the bones and liver and it increases in the brain and lungs [28, 29]. This distribution was not found in our cohort, we found a high rate of bone metastases (16.13%), a low rate of visceral metastases: 3.26% hepatic and 0.98% pulmonary. Cerebral metastases were not detected. The treatment of CSTN is based on surgery, radiotherapy and chemotherapy. Because of their high mitotic index, these tumors are more chemosensitive, especially to anthracyclines and taxanes [30]. The main objective of neoadjuvant chemotherapy is to decrease tumor size and to increase the proportion of conservative breast surgery, it is proposed for inflammatory or locally advanced tumors, inoperable from the outset and for operable tumors but voluminous, not accessible to conservative treatment [31]. In neoadjuvant situations, TNBC have a high rate of pCR compared to other subtypes. The pCR is defined by the absence of residual invasive cancer after neoadjuvant chemotherapy both in the breast and in the lymph nodes. Thus, it is well demonstrated that the pCR to neoadjuvant chemotherapy is associated with an improvement in survival [29, 30, 32, 33]. The rate of pCR varies from 22% to 36% [10,11, 29, 30]. The treatment of TNBC is paradoxical, despite their chemosensitivity, the prognosis of the TNBC remains poor due to the aggressive biological characteristics and the appearance of an early chemoresistance [34]. In our study, 34 women underwent neoadjuvant chemotherapy followed by surgery. The majority of patients received anthracyclines associated with taxanes. The pCR rate was 29%, which is consistent with the literature. Death occurs mostly in the first five years, especially during the absence of complete pathological response (pCR) after neoadjuvant therapy. All deaths from triple-negative breast cancer occur within 10 years of diagnosis [14]. In our study the survival rate for all patients at 5 years was 45.2%, this rate is significantly lower than that reported in several studies [10-12, 14, 16, 29]. We suggest that this is due to the tardy diagnosis and to the advanced age of the patients (Figure 1) so they are less likely to respond to systemic treatment.

## Conclusion

TNBC is aggressive and associated with young age, high tumor size and a high rate of visceral metastasis. This descriptive study shows that most of TNBC characteristics in the east of Algeria are consistent with literature data. However, it is more common in menopausal women and associated with lower rate of visceral metastasis and lower overall survival at 5 years. It is indispensable to sensitize women to the importance of early diagnosis of breast cancer and to organize detection campaigns in all the structures of the ministry of health to increase the chances of healing and improve the survival of patients. The identification of genes predisposing TNBC is necessary to understand the complexity of this type and to develop therapies to improve survival of patients. Characterization of target genes will be undertaken to determine the genetic profile of our population.

### What is known about this topic

- TNBC is a heterogeneous group characterized by a large tumor size and by a high rate of visceral metastasis;
- It generally occurs in younger women, less than 50 years;
- It is more frequent in specific ethnic groups (African and Hispanic women).

### What this study adds

- First descriptive study of TNBC in Algeria;
- TNBC is also common in menopausal women;
- Visceral metastasis are not always frequent in this type.

## Competing interests

The authors declare no competing interests.
